# Food Environment After Implementation of a Healthy Checkout Policy

**DOI:** 10.1001/jamanetworkopen.2024.21731

**Published:** 2024-08-08

**Authors:** Jennifer Falbe, Samantha Marinello, Ethan C. Wolf, Sarah Solar, Lisa M. Powell

**Affiliations:** 1 Human Development and Family Studies Program, Department of Human Ecology, University of California, Davis; 2 Health Policy and Administration, School of Public Health, University of Illinois Chicago; 3 Public Health Nutrition Program, Community Health Sciences, School of Public Health, University of California, Berkeley

## Abstract

**Importance:**

In March 2021, Berkeley, California, became the world’s first jurisdiction to implement a healthy checkout policy, which sets nutrition standards for foods and beverages in store checkouts. This healthy checkout ordinance (HCO) has the potential to improve customers’ dietary intake if stores comply by increasing the healthfulness of foods and beverages at checkouts.

**Objectives:**

To compare the percentage of checkout products that were HCO compliant and that fell into healthy and unhealthy food and beverage categories before and 1 year after HCO implementation in Berkeley relative to comparison cities.

**Design, Setting, and Participants:**

In this cohort study in which Berkeley implemented an HCO and other cities did not, a difference-in-differences analysis was conducted of 76 258 product facings at checkouts of 23 stores in Berkeley and 75 stores in 3 comparison cities in California. Data were collected in February 2021 (approximately 1 month before implementation of the HCO) and 1 year later in February 2022 and analyzed from October 2023 to May 2024.

**Exposure:**

The HCO, which permits only the following products at checkouts in large food stores: nonfood and nonbeverage products, unsweetened beverages, and foods with 5 g or less of added sugars per serving and 200 mg or less of sodium per serving in the following categories: sugar-free gum and mints, fruit, vegetables, nuts, seeds, legumes, yogurt or cheese, and whole grains.

**Main Outcomes and Measures:**

A product facing’s (1) HCO compliance and (2) category, including healthy compliant categories and unhealthy noncompliant categories, determined using a validated photograph-based tool to assess product characteristics.

**Results:**

Of the 76 258 product facings at store checkouts, the percentage that were HCO compliant increased from 53% (4438 of 8425) to 83% (5966 of 7220) in Berkeley, a 63% increase relative to comparison cities (probability ratio [PR], 1.63; 95% CI, 1.41-1.87). The percentage of food and beverage checkout facings that were HCO compliant increased in Berkeley from 29% (1652 of 5639) to 62% (2007 of 3261), a 125% increase relative to comparison cities (PR, 2.25; 95% CI, 1.80-2.82). The percentage of Berkeley food and beverage facings consisting of candy, sugar-sweetened beverages, and other sweets significantly decreased (candy: from 30% [1687 of 5639] to 6% [197 of 3261]; PR, 0.21; 95% CI, 0.10-0.42; sugar-sweetened beverages: from 11% [596 of 5639] to 5% [157 of 3261]; PR, 0.41; 95% CI, 0.23-0.75; other sweets: from 7% [413 of 5639] to 3% [101 of 3261]; PR, 0.37; 95% CI, 0.15-0.88), while the percentage consisting of unsweetened beverages (from 4% [226 of 5639] to 19% [604 of 3261]; PR, 4.76; 95% CI, 2.54-8.91) and healthy foods (from 6% [350 of 5639] to 20% [663 of 3261]; PR, 2.90; 95% CI, 1.79-4.72) significantly increased.

**Conclusions and Relevance:**

This cohort study of the first healthy checkout policy found substantial improvements in the healthfulness of food environments at checkouts 1 year after implementation of the policy. These results suggest that healthy checkout policies have the potential to improve the healthfulness of store checkouts.

## Key Points

**Question:**

Did the healthfulness of food and beverage products placed in Berkeley, California, store checkouts improve approximately 1 year after the implementation of a mandatory healthy checkout policy relative to cities without a healthy checkout policy?

**Findings:**

In a cohort study of 76 258 checkout product facings in 98 stores, the percentage of facings that were compliant with the healthy checkout policy increased by 63% in Berkeley relative to comparison cities, with 83% of Berkeley checkout products being compliant after policy implementation. The percentage of Berkeley food and beverage checkout facings that consisted of candy, sugar-sweetened beverages, and other sweets significantly decreased, while the percentage consisting of unsweetened beverages and healthy food significantly increased.

**Meaning:**

These results suggest that healthy checkout policies have the potential to improve the healthfulness of store checkouts.

## Teaser

This cohort study compares the percentage of checkout food and beverage products that were compliant with a healthy checkout ordinance before and 1 year after policy changes, relative to comparison cities, and assesses checkout products that fell into healthy and unhealthy food categories.

## Introduction

In March 2021, Berkeley, California, became the world’s first jurisdiction to implement a healthy checkout policy, which sets nutrition standards for foods and beverages placed in store checkout areas.^1,2^ The Berkeley healthy checkout ordinance (HCO) permits only the following foods and beverages at checkout: beverages without sweeteners (caloric or noncaloric) and foods with 5 g or less of added sugars per serving and 200 mg or less of sodium per serving in the following categories: sugar-free gum and mints, fruit, vegetables, nuts, seeds, legumes, yogurt or cheese, and whole grains. The policy applies to all checkouts in large stores (>2500 ft^2^ [>232 m^2^]) that sell at least 25 linear feet (7.6 linear meters) of food. Outreach to stores about the policy occurred in 2020-2021. The policy was enforceable starting January 2022, and inspections began in 2023. After Berkeley, the UK^3^ and Perris, California,^4^ implemented similar policies.

The stated rationale for the HCO was that consumption of excess sugar and sodium increases risk of diabetes, high blood pressure, and stroke; that choices are affected by placement of foods and beverages at checkouts; and that it is in the interest of the health and welfare of those in Berkeley that large stores offer healthy options and do not actively encourage the purchase of unhealthy foods.^1^

The HCO has the potential to improve dietary intake because (1) an estimated 70% of food and beverage products at store checkouts are unhealthy (eg, sugar-sweetened beverages [SSBs], candy)^2^; (2) most price promotions at checkouts are for unhealthy foods and beverages^5^; and (3) as the only place all customers must pass through, the checkout is known to induce impulse purchases.^6,7^ In a national US study, 36% of adults reported buying a food or beverage found at checkout during their last grocery trip.^8^ Indicative of the HCO’s potential to improve the healthfulness of consumer purchases is evidence that voluntary checkout standards focused on limiting candy and sweets^9^ were associated with a sustained reduction in purchases of sweets and salty snacks.^10^ The HCO may also promote nutrition equity by reducing neighborhood socioeconomic and racial and ethnic disparities in exposure to unhealthy foods and beverages at checkouts.^11^

For a healthy checkout policy to improve consumer purchases and dietary intake, it must first improve the healthfulness of the food environment at checkout through store compliance. To our knowledge, there have been no evaluations to date of the impact of a mandatory healthy checkout policy on food environments. We sought to compare the percentage of checkout food and beverage products that were HCO compliant before and 1 year after HCO policy changes, as well as the percentage of checkout products that fell into specific healthy and unhealthy food categories in Berkeley relative to comparison cities.

## Methods

### Design and Sample

In a natural experiment in which Berkeley implemented an HCO and other cities did not, we conducted a cohort study of product facings available at store checkouts. The eTable in Supplement 1 shows the analytic store sample: a census of 23 Berkeley stores initially identified by policy proponents as subject to the ordinance and 75 stores from comparison cities open in February 2021 (≤1 month before implementation) and February 2022 (approximately 1 year after implementation). The Berkeley stores included all of the city’s supermarkets, chain specialty food stores, chain drugstores, and mass merchandisers; 2 of the city’s 3 dollar stores (1 dollar store was too small to be subject to the HCO); and all independent grocery stores larger than 2500 ft^2^ (232 m^2^). The only store subject to the ordinance that was not included in the sample is the 1 convenience store in Berkeley that is larger than 2500 ft^2^ (232 m^2^). This convenience store was not included because the sampling strategy was based on early discussions among policy proponents that suggested convenience stores as a category would not be subject to the HCO. However, ultimately, store size and linear measurement of food displayed were used as criteria for store inclusion in the HCO. Institutional review board review was waived as this study was determined to be nonhuman subjects research by the University of California, Davis institutional review board. This study followed the Strengthening the Reporting of Observational Studies in Epidemiology (STROBE) reporting guideline.

The comparison California cities of Davis, Oakland, and Sacramento were chosen based on having urbanicity, racial and ethnic diversity, and economic indicators similar to Berkeley.^12^ Comparison stores in each city were selected using stratified random sampling to match Berkeley stores by chain and type when possible (eTable in Supplement 1).^2^ However, if a comparison city had an insufficient number of matching stores by type, we sampled additional stores of that type from other comparison cities. When possible, we oversampled within store type in comparison cities to buffer against store closures or refusals.

Of 24 Berkeley stores and 88 comparison stores initially sampled, 10 comparison stores could not be assessed because 5 refused data collection, 4 did not sell products at checkout, and 1 was perceived as unsafe. This left a sample of 102 stores assessed during the preimplementation period. Of these 102 stores, 1 Berkeley store and 3 comparison stores closed prior to the postimplementation period, leaving a final sample of 98 stores (eTable in Supplement 1).

Within each store, we sampled all checkouts or 3 randomly selected checkouts if a store had more than 3 checkouts.^2^ A checkout was defined as any area accessible to customers within a 3-ft (0.9-m) distance to any register or any area designated or used primarily to wait in line to make a purchase, including the checkout endcap.^1,2^

We assessed a census of product facings accessible to consumers^2^ at each sampled checkout before and after HCO implementation. A product facing was defined as each product that faced customers but did not include products stacked behind it.^2^ If the original checkout was unavailable after HCO implementation, a replacement checkout in that store was randomly sampled.

### Outcomes and Measures

The study outcomes included a product facing’s (1) HCO compliance and (2) food and beverage category. Healthy checkout ordinance compliance was defined by the ordinance as including only unsweetened beverages, foods in specific categories (ie, gum and mints with no added sugars, fruit, vegetables, nuts, seeds, legumes, yogurt or cheese, and whole grains) that contained 5 g or less of added sugars per serving and 200 mg or less of sodium per serving, and nonfood and nonbeverage products. Within this category, facings were additionally classified as a compliant food or beverage or as a nonfood and nonbeverage facing.

The outcome of food and beverage category included healthy categories permissible by the HCO and unhealthy noncompliant categories.^2^ Healthy categories permissible by the HCO included gum and mints with no added sugars, unsweetened beverages, and healthy foods (ie, nuts and seeds, fruits and vegetables, and legumes, cheese, and whole grains) containing 5 g or less of added sugars per serving and 200 mg or less of sodium per serving. Unhealthy noncompliant categories included SSBs, nonnutritively sweetened beverages, candy, salty snacks (eg, chips, crackers, pretzels, and dried meat), other sweets (eg, cookies, fruit snacks, pudding [not including candy]), and other noncompliant products (ie, any other nonpermissible food category [eg, white bread, sauces, and oil] or foods in HCO-permissible categories that exceeded HCO limits on added sugar or sodium).

To collect data on product facings at checkout and to classify them by compliance and category, trained data collectors photographed and assessed characteristics of each facing using the Store Checkout Tool (SCOUT).^13^ SCOUT entails taking wide-angle and close-view digital photographs of every product facing in sampled checkout areas so that characteristics of each product (eg, brand, flavor, and size) can be coded from photographs. SCOUT has demonstrated high interrater reliability, with mean κ = 0.95 and mean intraclass correlation coefficient greater than 0.99. Nutritional composition and ingredients were then coded from manufacturers’ websites (or, if not available, from retailer websites and publicly accessible databases) and merged with facing data.^2^ After excluding 3796 of 80 054 out-of-stock or obscured facings and facings without retrievable nutritional information (5%), the analytic sample comprised 76 258 facings, of which 50 061 (66%) were food and beverage facings.

### Statistical Analysis

Statistical analysis was conducted from October 2023 to May 2024 using Stata/MP, version 18.0 (StataCorp LLC). The unit of analysis was the product facing. Summary statistics describe counts and percentages of facings by compliance and food and beverage category in Berkeley and comparison cities during the preimplementation and postimplementation periods. The percentages of product facings that were compliant were calculated among (1) all checkout product facings and (2) only food and beverage checkout facings.

To estimate preimplementation to postimplementation changes in the percentages of products that were compliant and that fell into specific food and beverage categories in Berkeley relative to comparison cities, difference-in-differences Poisson regression models with robust SEs clustered on store were used. These difference-in-differences regressions were estimated among (1) all checkout product facings and among (2) only food and beverage checkout facings. Specifically, models of the following form were estimated:

log(*Y_ist_) _ = _  β*_0_ + β_1_*Post_t_ + β*_2_*Berkeley_s_ + β*_3_(*Post_t_* × *Berkeley_s_*) + *ε_ist_*

where *Y_ist_* is the outcome for product facing *i* in site *s* at time *t*. *Post_t_* is an indicator for the postimplementation period, and *Berkeley_s_* is an indicator for being in Berkeley. The exponentiated coefficient *β_3_* represents the difference-in-differences estimate—the probability ratio (PR), which is a ratio of ratios indicating the change in the probability that facings were (1) compliant or (2) fell in a product category in Berkeley relative to comparison cities.

Also, although the distribution of store types was similar between Berkeley and comparison cities, due to modest differences (eg, chain drug stores comprised 30% of Berkeley stores [7 of 23] vs 32% of comparison stores [24 of 75]; eTable in Supplement 1), we conducted sensitivity analyses adjusting for store type. Last, to describe compliance by store type, we calculated the percentage of all checkout facings and food and beverage facings that were compliant before and after HCO implementation within each store type.

## Results

**Figure 1.**
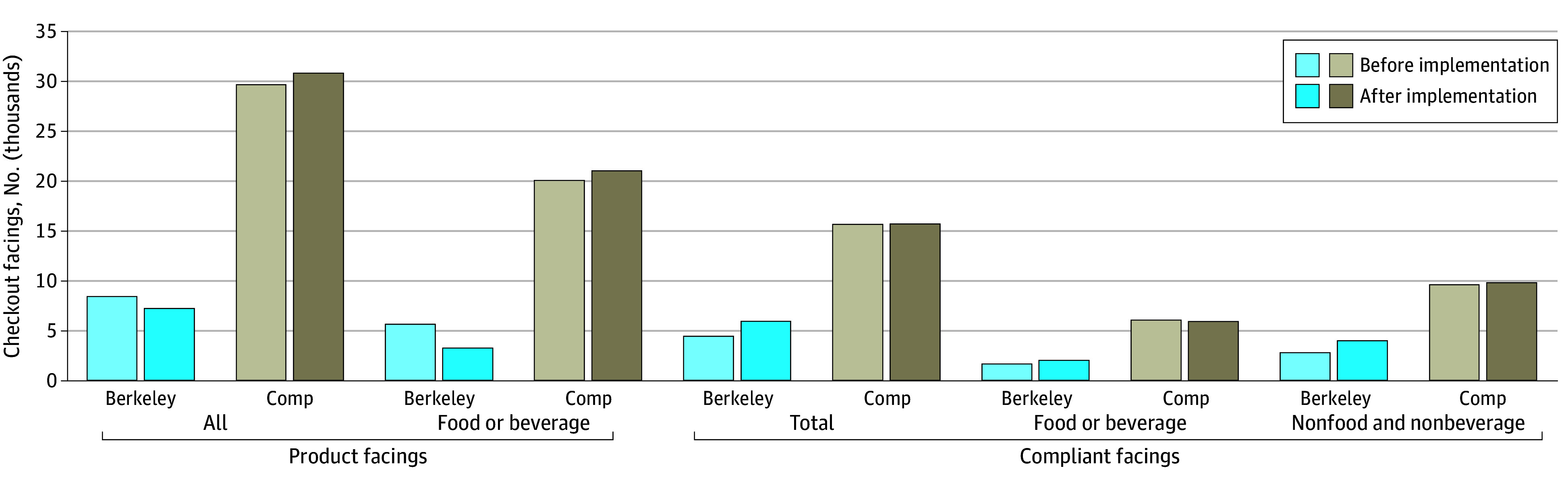
Number of All Checkout Product Facings, Food and Beverage Checkout Facings, and Compliant Checkout Facings Before and After Implementation of the Healthy Checkout Ordinance in Berkeley and Comparison Cities (Comp) Compliant facings included both compliant food and beverage facings and nonfood and nonbeverage facings. The total sample size included 76 258 checkout product facings, of which 50 061 were food and beverage facings.

Figure 1 shows the counts of all product facings, food and beverage facings, and compliant product facings before and 1 year after HCO implementation in Berkeley and comparison cities. The number of all checkout product facings and the number of compliant checkout facings were stable over time in the comparison cities. However, in Berkeley, the number of all checkout product facings decreased between the preimplementation and postimplementation periods due to a substantial decrease in the number of food and beverage facings at checkout. Over this 1-year period, the number of compliant food and beverage facings and nonfood and nonbeverage facings increased in Berkeley checkouts.

**Figure 2.**
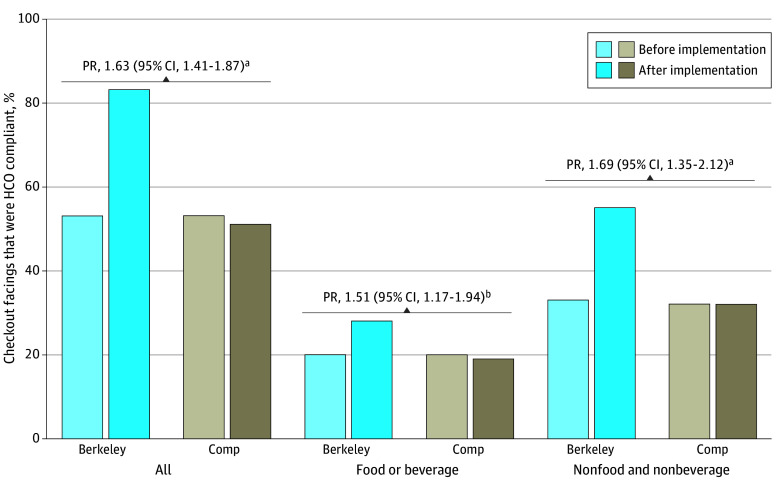
Percentage of All 76 258 Checkout Facings That Were Compliant With the Healthy Checkout Ordinance Before and After Implementation and Probability Ratios (PRs) Comparing Changes in Berkeley With Those in Comparison Cities (Comp) Compliant facings included both compliant food and beverage facings and nonfood and nonbeverage facings. Difference-in-differences PRs were calculated using Poisson models with robust SEs clustered on store that regressed compliance on a postimplementation indicator, a Berkeley indicator, and a postimplementation × Berkeley interaction term. ^a^*P* < .001. ^b^*P* < .01.

Figure 2 shows the main results for the percentage of all checkout facings that were HCO compliant before and 1 year after implementation in Berkeley and comparison cities as well as difference-in-differences PRs. The percentage of all facings that were HCO compliant increased from 53% (4438 of 8425) to 83% (5966 of 7220) in Berkeley, a 63% increase in compliance in Berkeley relative to the comparison cities (PR, 1.63; 95% CI, 1.41-1.87), where there was little change (from 53% [15 691 of 29 737] to 51% [15 723 of 30 876]). This increase in compliance in Berkeley was due to increases in the percentage of all checkout facings consisting of compliant food and beverage facings (from 20% [1652 of 8425] to 28% [2007 of 7220]; PR, 1.51; 95% CI, 1.17-1.94) and nonfood and nonbeverage facings (from 33% [2786 of 8425] to 55% [3959 of 7220]; PR, 1.69; 95% CI, 1.35-2.12) compared with virtually no change in comparison cities.

**Figure 3.**
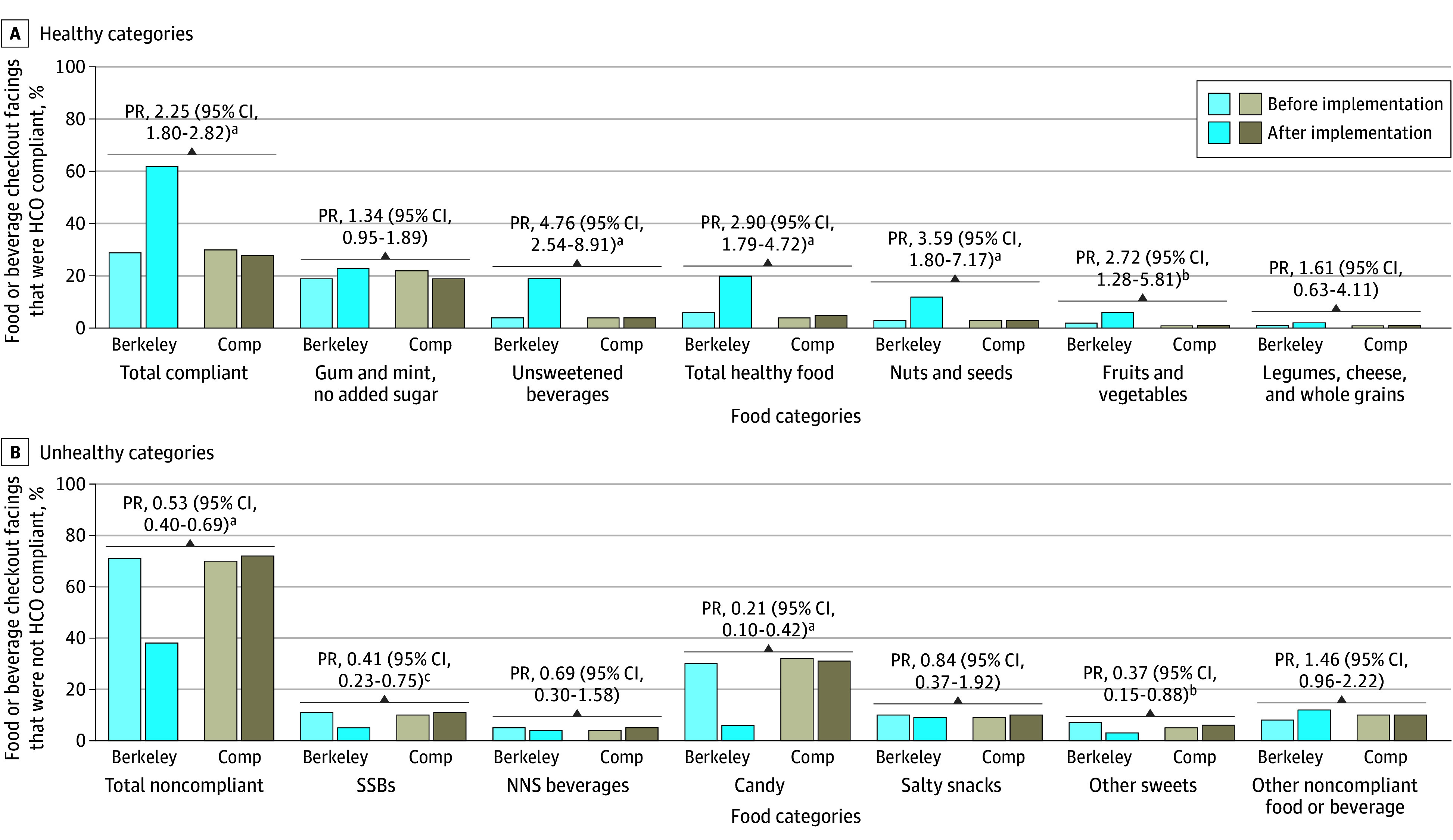
Percentage of 50 061 Food and Beverage Checkout Facings That Were Healthy Checkout Ordinance (HCO) Compliant Before and After Implementation and Probability Ratios (PRs) Comparing Changes in Berkeley With Comparison Cities (Comp) Models were restricted to food and beverage product facings only (n = 50 061). Difference-in-differences PRs were calculated using Poisson models with robust SEs clustered on store that regressed compliance on a postimplementation indicator, a Berkeley indicator, and a postimplementation × Berkeley interaction term. “Total healthy food” includes nuts and seeds, fruits and vegetables, and legumes, cheese, and whole grains. “Other sweets” did not include candy. “Other noncompliant food or beverage” included any other nonpermissible category (eg, white bread, oil) or foods in HCO-permissible categories that exceeded HCO limits for added sugar or sodium. NNS indicates nonnutritively sweetened; SSBs, sugar-sweetened beverages. ^a^*P* < .001. ^b^*P* < .05. ^c^*P* < .01.

Figure 3 shows the main results when only food and beverage checkout facings were considered, for which there was little to no change in comparison cities. Among only food and beverage checkout facings, compliance increased in Berkeley from 29% (1652 of 5639) to 62% (2007 of 3261), a 125% increase in Berkeley relative to the comparison cities (PR, 2.25; 95%, CI, 1.80-2.82; Figure 3A). This increase was due to large increases in Berkeley in the percentage of food and beverage facings consisting of unsweetened beverages (from 4% [226 of 5639] to 19% [604 of 3261]; PR, 4.76; 95% CI, 2.54-8.91) and healthy foods (from 6% [350 of 5639] to 20% [663 of 3261]; PR, 2.90; 95% CI, 1.79-4.72), particularly nuts and seeds (from 3% [173 of 5639] to 12% [386 of 3261]; PR, 3.59; 95% CI, 1.80-7.17) and fruits and vegetables (from 2% [94 of 5639] to 6% [199 of 3261]; PR, 2.72; 95% CI, 1.28-5.81).

The increase in compliance in Berkeley also meant that the percentage of food and beverage checkout facings that consisted of unhealthy, noncompliant products decreased by about half (Figure 3B), which resulted from large decreases in candy (from 30% [1687 of 5639] to 6% [197 of 3261]; PR, 0.21; 95% CI, 0.10-0.42) and SSBs (from 11% [596 of 5639] to 5% [157 of 3261]; PR, 0.41; 95% CI, 0.23-0.75), and to a lesser extent, other sweets (from 7% [413 of 5639] to 3% [101 of 3261]; PR, 0.37; 95% CI, 0.15-0.88). The eFigure in Supplement 1 shows before-and-after photos of select Berkeley checkouts to illustrate the improvements in the healthfulness of Berkeley checkouts.

**Figure 4.**
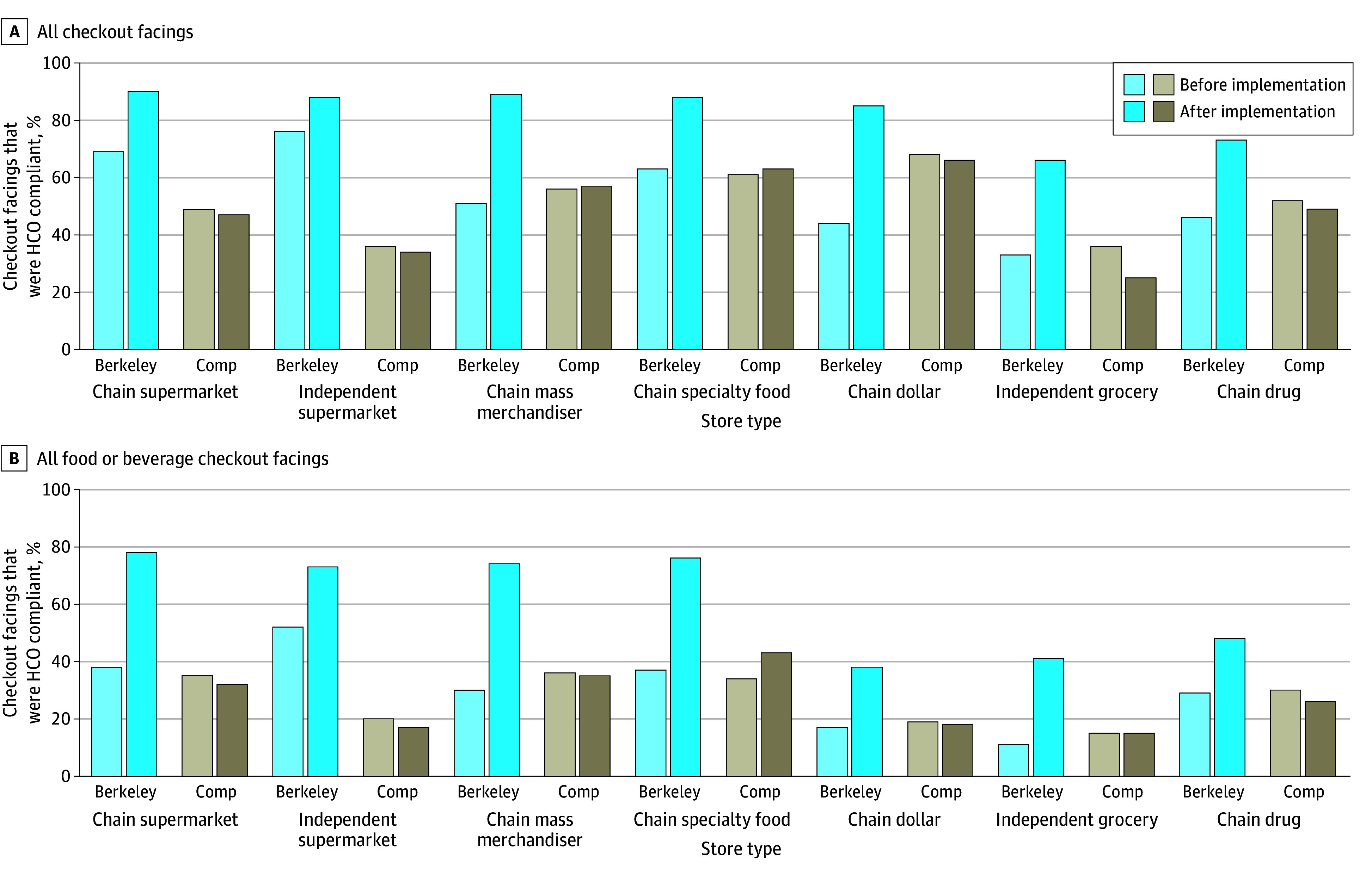
Percentage of Checkout Facings by Store Type That Were Healthy Checkout Ordinance Compliant Before and After Implementation in Berkeley and Comparison Cities (Comp) A, All checkout facings (N = 76 258). B, Food or beverage checkout facings (n = 50 061).

Figure 4 shows the following by store type: percentage of all checkout facings (Figure 4A) and percentage of food and beverage checkout facings (Figure 4B) that were HCO compliant in Berkeley and comparison cities during the preimplementation and postimplementation periods. Across Berkeley store types, the proportion of all checkout facings and food and beverage facings that were compliant increased. Among all checkout facings, the largest percentage point increases in compliance in Berkeley occurred in chain dollar stores (from 44% [564 of 1280] to 85% [693 of 812]), mass merchandisers (from 51% [652 of 1283] to 89% [1016 of 1138]), independent grocery stores (from 33% [141 of 429] to 66% [259 of 395]), and chain drug stores (from 46% [1336 of 2890] to 73% [1543 of 2117]). Among only food and beverage checkout facings, the largest percentage point increase in compliance occurred in chain mass merchandisers (from 30% [266 of 897] to 74% [342 of 464]), chain supermarkets (from 38% [205 of 544] to 78% [472 of 603]), chain specialty food stores (from 37% [178 of 479] to 76% [259 of 342]), and independent grocery stores (from 11% [35 of 323] to 41% [95 of 231]). In comparison, the proportion of facings that were compliant in comparison cities remained relatively stable over time by store type (except independent grocery stores).

During the postimplementation period in Berkeley, most checkout facings were compliant across store type, with highest compliance observed in chain supermarkets (90% [1241 of 1372]), mass merchandisers (89% [1016 of 1138]), independent supermarkets (88% [629 of 718]), chain specialty food stores (88% [585 of 668]), and dollar stores (85% [693 of 812]), with somewhat lower compliance in chain drug stores (73% [1543 of 2117]) and independent grocery stores (66% [259 of 395]). However, postimplementation compliance among only food and beverage facings in Berkeley was more heterogeneous by store type, with highest compliance among chain supermarkets (78% [472 of 603]), chain specialty food stores (76% [259 of 342]), chain mass merchandisers (74% [342 of 464]), and independent supermarkets (73% [238 of 327]) but substantially lower compliance in Berkeley chain drug stores (48% [528 of 1102]), independent grocery stores (41% [95 of 231]), and chain dollar stores (38% [73 of 192]).

## Discussion

Approximately 1 year after implementation (although just 1 month after Berkeley’s HCO became enforceable), there were large improvements in the healthfulness of products stocked at store checkouts. Relative to comparison cities, Berkeley experienced a 63% increase in the percentage of all checkout facings that were HCO compliant, from 53% before implementation to 83% after implementation. When only food and beverage checkout facings were considered, Berkeley experienced an even greater increase—125%—in healthfulness of facings relative to comparison cities, from 29% to 62%. Stores replaced unhealthy food and beverage checkout facings, particularly SSBs and candy, with nonfood and nonbeverage products, unsweetened beverages, and healthy foods (nuts, seeds, fruits, and vegetables).

The highest postimplementation compliance in Berkeley by store type was observed in chain and independent supermarkets, chain mass merchandisers, and chain specialty food stores (88%-90% compliance among all facings and 73%-78% compliance among food and beverage facings). These store types, especially supermarkets, are also where US households most frequently shop^14^ and from which most calories are purchased in the US.^15^ Thus, the higher compliance in these stores means that consumer exposure to healthier product facings in store checkouts may be even higher in Berkeley than the overall compliance percentages would indicate.

In contrast, Berkeley chain dollar, independent grocery, and chain drug stores exhibited a markedly higher postimplementation compliance among all checkout facings (66%-85%) than among only food and beverage facings (38%-48%), indicating that these stores were stocking relatively more nonfood and nonbeverage products than healthy foods at checkout compared with other store types. However, in all store types, the percentage of food and beverage facings that were compliant increased in Berkeley but to varying degrees.

The high level of HCO compliance observed in Berkeley was attained after outreach to stores about the policy had been conducted but prior to enforcement, which is described in the ordinance as including inspections and citations, including fines (eg, paying for costs associated with investigating complaints). Initial outreach and technical assistance around the HCO was provided to stores between December 2020 and November 2021 by a youth-focused, community-based organization that received a grant from the city to work on implementation as a result of a community grantmaking process established by Berkeley’s SSB excise tax ordinance.^16^ Healthy checkout ordinance compliance, and therefore the healthfulness of store checkout environments, may increase further (especially in independent grocery and chain drug stores) after city inspections are completed, which began in 2023.

Because this is the first evaluation of a healthy checkout policy, to our knowledge, there were no similar studies for comparison. However, the level of compliance observed herein exceeds that observed in other studies of mandatory healthy retail or restaurant policies. For example, an evaluation of the 2015 Minneapolis Staple Food Ordinance, which required food stores to stock minimum quantities of healthy foods and beverages in specific categories, found that there was no difference in change between stores in Minneapolis, Minnesota, and comparison stores in St. Paul, Minnesota.^17^ Furthermore, by 2017, compliance among sampled stores was fairly low, with only 10% of stores being fully compliant and only 51% being mostly compliant. Likewise, evaluations of healthy beverage default laws regulating restaurant children’s meals have found low postpolicy compliance (eg, 6%-41%) and/or no significant difference in prepolicy to postpolicy change between jurisdictions subject to the policy and comparison jurisdictions.^18,19,20^

Despite the Staple Food Ordinance and healthy beverage default laws having enforcement mechanisms similar to the Berkeley HCO, including inspections, warnings, and fines, competing public health priorities and limited capacity of enforcement staff may have contributed to low compliance with these other policies.^17,18,19^ Future research is needed to identify why there was relatively high compliance with the Berkeley HCO. However, prior research on other policies indicates that compliance may be higher among chain retailers, which comprised 74% of the Berkeley sample, when a policy is simple, when technical assistance is provided, and if retailers are accustomed to regulation.^16,21,22^ It is possible that the messenger of the technical assistance matters as well, which in Berkeley was a youth-focused community organization. Also, a qualitative study was conducted prior to the October 2022 implementation of a recent UK policy (which has not yet been evaluated) that restricts products high in saturated fat, sugars, and salt from prominent places in stores; the study assessed affected parties’ perceptions of government support that could help with policy implementation and compliance, identifying a product compliance calculator, refinement of the legislation, and greater support for smaller businesses and local authorities as potentially important.^3^

### Strengths and Limitations

Study strengths include using a reliable and objective tool to assess product facing characteristics,^13^ comparison cities to provide evidence that changes observed in Berkeley were not due to secular trends, and a large sample size of product facings (>76 000) linked with their nutritional information. Study limitations include not having monthly data to understand any potential fluctuations in the healthfulness of checkout environments around various holidays and not including the 1 convenience store in Berkeley that was subject to the HCO policy.

## Conclusions

This cohort study found that, just 1 year after implementation of the Berkeley HCO, the healthfulness of checkout environments improved substantially through high levels of store compliance. Berkeley stores increased the percentage of checkout facings that were compliant food and beverage and nonfood and nonbeverage products. The percentage of food and beverage checkout facings consisting of candy, SSBs, and other sweets significantly decreased, while the percentage consisting of unsweetened beverages and healthy food significantly increased in Berkeley relative to comparison cities. Research is needed on long-term associations of healthy checkout policies with store environments and consumer behavior.

## Notes

Supplement 1. **eTable.** Store Sample in Berkeley and Comparison Cities **eFigure.** Before-and-After Photos of Select Berkeley Checkouts Assessed in 2021 and 2022

Supplement 2.
Data Sharing Statement
