# Supplementary material for: Food Environment After Implementation of a Healthy Checkout Policy
Source: JAMA Netw Open. 2024 Aug 8;7(8):e2421731. doi: 10.1001/jamanetworkopen.2024.21731 (PMC11310826; doi:10.1001/jamanetworkopen.2024.21731)
Supplement: Supplement 1. — eTable. Store Sample in Berkeley and Comparison Cities eFigure. Before-and-After Photos of Select Berkeley Checkouts Assessed in 2021 and 2022 [file jamanetwopen-e2421731-s001.pdf]

Supplementary Online Content

Falbe J, Marinello S, Wolf EC, Solar S, Powell LM. Food environment after implementation of a healthy checkout policy. *JAMA Netw Open.* 2024;7(8):e2421731. doi:10.1001/jamanetworkopen.2024.21731

eTable. Store Sample in Berkeley and Comparison Cities

eFigure. Before-and-After Photos of Select Berkeley Checkouts Assessed in 2021 and 2022

This supplementary material has been provided by the authors to give readers additional information about their work.

© 2024 Falbe J et al. *JAMA Network Open.*

eTable. Store Sample in Berkeley and Comparison Cities

	Berkeley, n (%)	Comparison cities, n (%)				All, n (%)
**Store type**		**Davis**	**Oakland**	**Sacramento**	**Total comparison**	
**Chain dollar store**	2 (9%)	2	3	3	8 (11%)	11 (11%)
**Chain drug store**	7 (30%)	3	10	11	24 (32%)	33 (34%)
**Chain specialty food**	3 (13%)	1	3	4	8 (11%)	11 (11%)
**Chain supermarket**	3 (13%)	6	4	3	13 (17%)	15 (15%)
**Chain mass merchandiser**	2 (9%)	1	1	4	6 (8%)	8 (8%)
**Independent supermarket**	2 (9%)	2	2	3	7 (9%)	9 (9%)
**Independent grocery**	4 (17%)	2	3	4	9 (12%)	27 (28%)
**Total**	23	17	26	32	75	98

Note: Stratified random sampling was used to match stores in each comparison city to stores in Berkeley by chain and store type when possible. If a city had an insufficient number of matching stores by type, we sampled additional stores of that type from other comparison cities, and within the city with insufficient stores of that type, we sampled additional stores of other types. A total of 1 Berkeley and 3 comparison stores closed between pre- and post-implementation periods, reducing the sample from 102 to 98.

© 2024 Falbe J et al. *JAMA Network Open.*

eFigure. Before-and-After Photos of Select Berkeley Checkouts Assessed in 2021 and 2022

Before | **After**

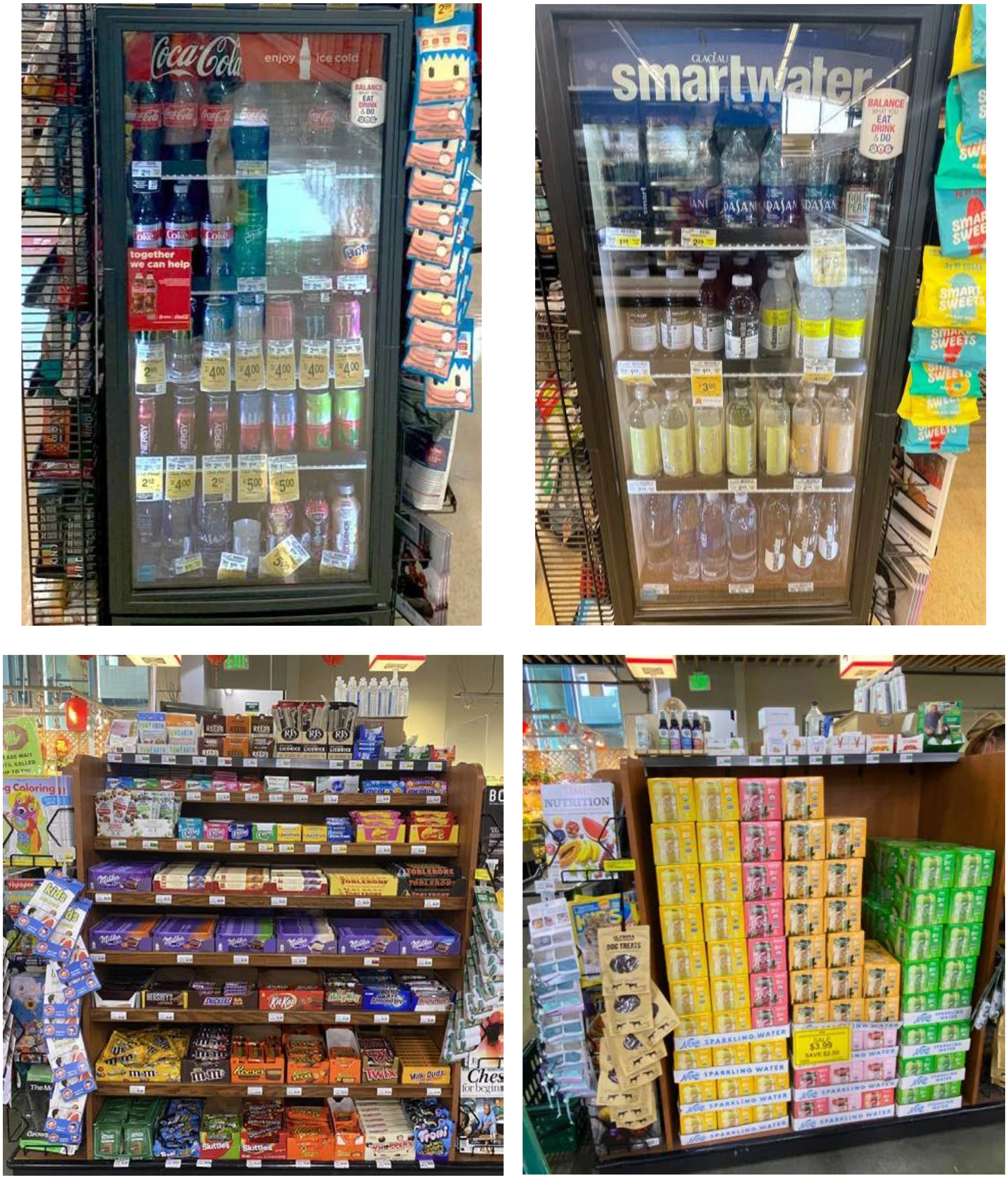

© 2024 Falbe J et al. *JAMA Network Open.*

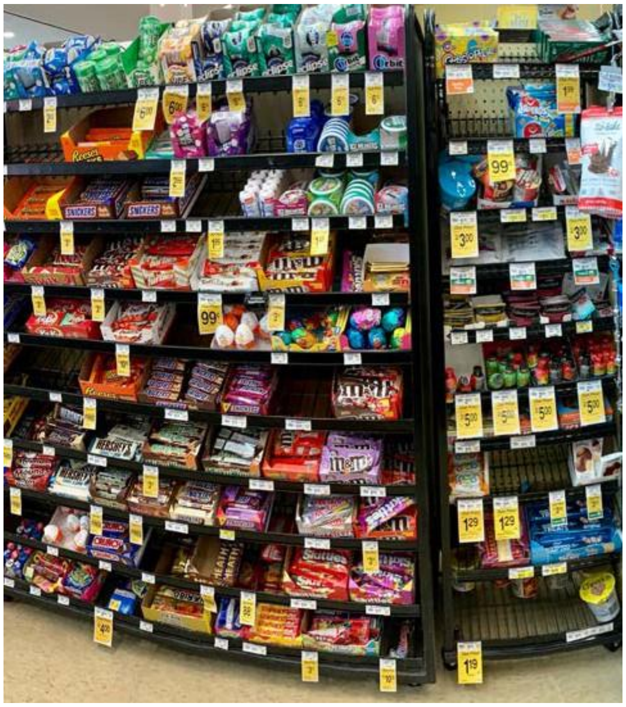

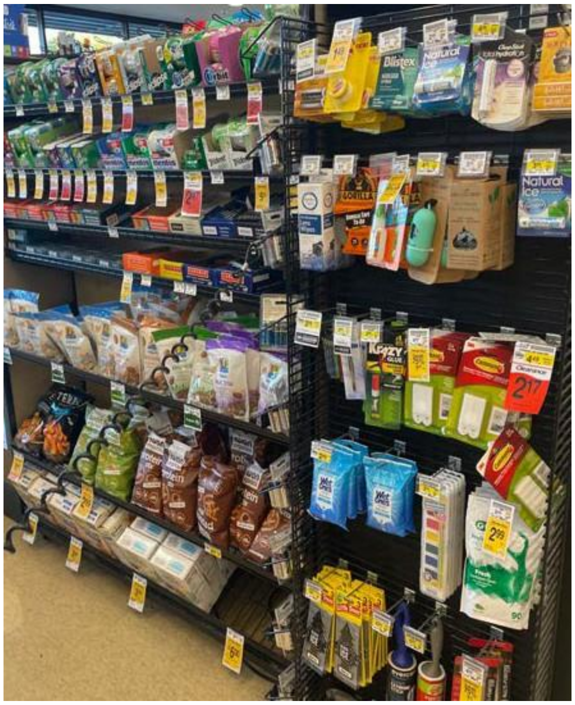

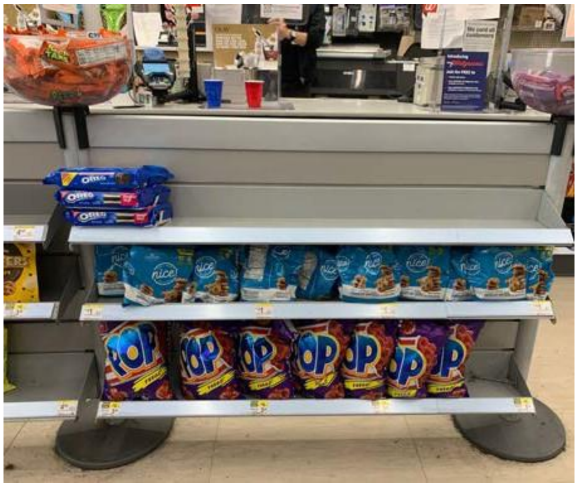

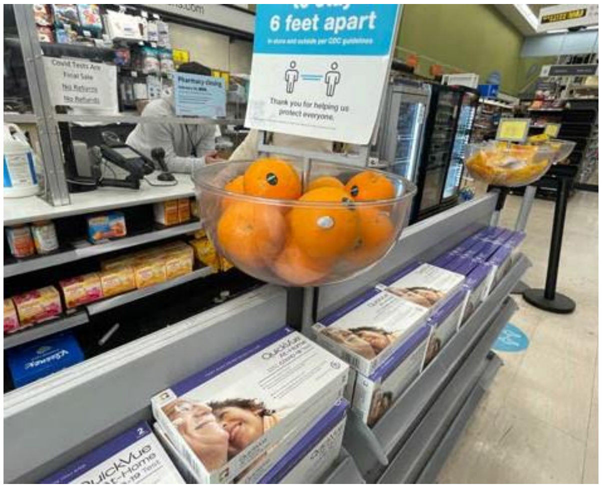

© 2024 Falbe J et al. *JAMA Network Open.*

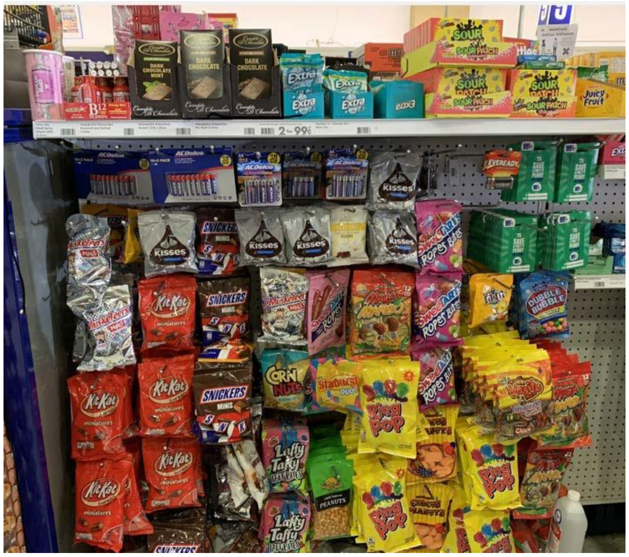

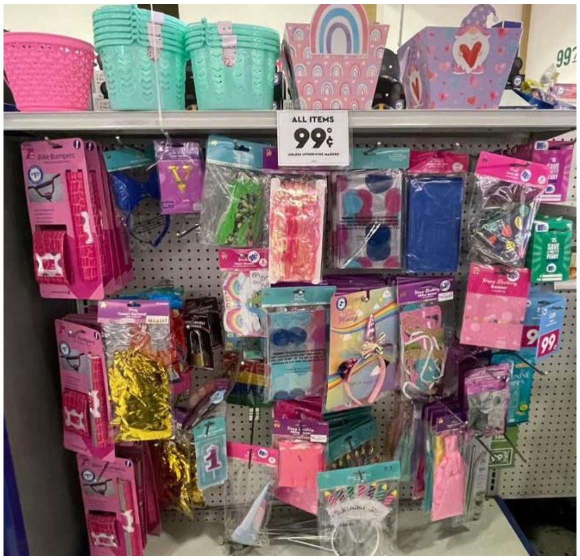

© 2024 Falbe J et al. *JAMA Network Open.*
