# Supplementary material for: Food Environment After Implementation of a Healthy Checkout Policy
Source: JAMA Netw Open. 2024 Aug 8;7(8):e2421731. doi: 10.1001/jamanetworkopen.2024.21731 (PMC11310826; doi:10.1001/jamanetworkopen.2024.21731)
Supplement: Supplement 2. — Data Sharing Statement [file jamanetwopen-e2421731-s002.pdf]

Data Sharing Statement

Falbe. Food Environment After Implementation of a Healthy Checkout Policy. *JAMA Netw Open*. Published August 08, 2024. doi:10.1001/jamanetworkopen.2024.21731

Data
Data available: Yes
Data types: Data (not involving human participants)
How to access data: The data will be made available under the auspices of the PI and with a data-sharing agreement that limits the use of data to only approved research for noncommercial purposes and prohibits the sharing of data with third parties. Data will be made available on the publication date of the final manuscript using the data.
When available: beginning date: 02-01-2030 (anticipated)

Supporting Documents
Document types: None

Additional Information
Who can access the data: Data will be made available under a data-sharing agreement that limits the use of data to only approved research for noncommercial purposes and prohibits the sharing of data with third parties. Data will be made available on the publication date of the final manuscript using the data.
Types of analyses: Noncommercial research
Mechanisms of data availability: Signed data access agreement
